# 684. Diagnostic Yield of Echocardiography in Coagulase Negative Staphylococcus Bacteremia

**DOI:** 10.1093/ofid/ofab466.881

**Published:** 2021-12-04

**Authors:** Sandhya Nagarakanti, Eliahu Bishburg, Alexis Okoh, Sagy Grinberg, Madhu Suryadevara

**Affiliations:** Newark Beth Israel Medical Center, Newark, NJ

## Abstract

**Background:**

Coagulase negative Staphylococcus (CoNS) bacteremia is a common clinical finding, but is less commonly associated with infective endocarditis (IE). Echocardiography (Echo) is utilized when clinicians suspect the diagnosis of IE. We sought to evaluate the utilization and yield of Echo in patients who had 1 or ≥ 2 (+) blood cultures (BC) for CoNS, and correlate Echo results with a diagnosis of IE.

**Methods:**

A retrospective review in a tertiary care hospital between 2013-2020. Patients with or without cardiac device, who had either 1 or ≥ 2 BC positive for CoNS and who underwent Echo were included. Modified Duke’s (MDC) criteria was used for the diagnosis of IE. Logistic regression was used to examine the association between BC positivity, device existence and the presence of a vegetation on Echo.

**Results:**

We included 116 patients, median age 58 (41-70) years, 64 (55%) women. Cardiac device was present in 69 (59%): Automated implantable cardioverter defibrillator in 49 (71%), pacemaker in 11(16%), ventricular assist device in four (6%), intra-aortic balloon pump in five (7%). CoNS isolated from 1 BC in 53(46%) patients and from ≥ 2 in 63(54%) patients. Trans- thoracic Echo (TTE) was performed in 42(36%), trans- esophageal Echo (TEE) in 39 patients (33.6%). Sequential Echo (TEE after TTE) was performed in 34 patients (29%). “Definite” IE was diagnosed in none, “possible” IE in 30 (26%), the diagnosis was “rejected” in 86 (74%). Vegetations were noted on device lead in 13(43%) and on valves in 17(57%). Overall yield in patients classified as “possible” IE (n=30) was similar in patients with device (n=26) to those without a device (n=4) (22% vs. 3%; p=0.149). For patients with 1 BC positive for CONS, the presence of a device was not associated with a positive Echo yield (OR, 95% C.I: 1.8 (0.3, 12.9); p=0.474). Patients who had ≥ 2 BC for CoNS had the same Echo yield with or without a cardiac device (15% vs. 24% p=0.243).

**Conclusion:**

In our medical center, patients with CoNS bacteremia, no patients had a “definite” diagnosis of IE. Yield of Echo was similar in patients with either one or ≥ 2 positive BC and there was no significant association with the presence of a device.

**Disclosures:**

**All Authors**: No reported disclosures

